# Mechanisms of microbiome-immune interactions in bladder cancer and targeted regulatory strategies

**DOI:** 10.3389/fimmu.2026.1920567

**Published:** 2026-09-17

**Authors:** Songhong Wu, Shi Fu, Hongjin Shi, Yicheng Gong, Peng Chen, Xiaoyue Zhang, Enyan Jiang, Haifeng Wang

**Affiliations:** Department of Urology, The Second Affiliated Hospital of Kunming Medical University, Kunming, China

**Keywords:** bladder cancer, epigenetic regulation, immune interactions, microbiota, therapeutic strategy

## Abstract

Bladder cancer (BC), a highly prevalent malignancy of the urinary tract, imposes a considerable clinical burden because of its high recurrence rates and a substantial risk of disease progression. Accumulating evidence suggests that microbiome dysbiosis is associated with the initiation and progression of BC through potentially bidirectional interactions. Recent advances in high-throughput sequencing technologies have facilitated a more comprehensive characterization of the urinary microbiome, thereby revealing its potential involvement in the pathogenesis of BC. This review summarizes the proposed mechanisms through which microbial communities may contribute to BC carcinogenesis, including chronic inflammation, microbial metabolite production, and epigenetic regulation. We further critically evaluate the established mechanisms of Bacillus Calmette–Guérin (BCG) immunotherapy—a standard intravesical immunotherapy for patients with intermediate- and high-risk non-muscle-invasive bladder cancer (NMIBC)—and examine the emerging application of microbiome profiling as a source of candidate noninvasive biomarkers and potential correlates or modulators of immune checkpoint inhibitor (ICI) response. Additionally, we elucidate the associations between microbiota heterogeneity and immunotherapy response, offering novel insights into microbiome-targeted therapeutic strategies for BC.

## 1 Introduction

Bladder cancer, a frequently occurring malignant neoplasm of the urinary system, demonstrates marked heterogeneity in its global epidemiological distribution. According to the 2020 Global Cancer Statistics report, approximately 573,000 new cases of bladder cancer are diagnosed annually, placing it tenth among malignancies worldwide (1, 2). The incidence rate in males (9.5 per 100,000) is markedly higher than that in females (2.4 per 100,000), yielding a male-to-female ratio of 3.96:1 (3). Currently, clinical treatment options for bladder cancer primarily include surgery and pharmacotherapy. Surgical intervention is associated with a relatively high postoperative recurrence rate, while chemotherapeutic approaches carry potential risks of drug toxicity and resistance. Microorganisms are closely associated with human tissues, often residing on mucosal surfaces and affecting a broad spectrum of physiological and pathological processes, including cellular homeostasis, metabolic regulation (4), inflammation (5), and immunity (6), thereby playing a crucial role in maintaining overall host balance (7).

In recent years, substantial advances have been achieved in clarifying the regulatory mechanisms underlying microbiome–host immune interactions within the tumor microenvironment. As a “dynamic metabolic organ,” the human commensal microbiome remodels host immune homeostasis through a bidirectional network of metabolic reprogramming and immune regulation, and its relationship with the onset and progression of bladder cancer has gradually shifted from phenomenological description to mechanistic understanding. The microbiome can also modulate immune checkpoint expression through epigenetic regulation (7). Furthermore, bladder cancer-specific experimental and clinical evidence suggests that sex-associated microbiota may shape the accumulation and function of immunosuppressive senescence-like neutrophils, potentially contributing to sex differences in bladder cancer incidence (8). These findings indicate that the microbiome may dynamically modulate the immunoediting process in bladder cancer through a multidimensional “metabolite–epigenetic–immune checkpoint” axis, thereby offering a theoretical foundation for targeted interventions.

This review centers on studies exploring the association between the microbiome and bladder cancer. The article systematically examines the mechanisms underlying microbiome–immune interactions in bladder cancer, outlines the potential contribution of the microbiome to tumor initiation and progression, highlights the carcinogenic or protective functions of specific microbial species, and examines how the microbiome influences therapeutic outcomes. This review is intended to offer a comprehensive perspective and deeper insights into this field, thereby facilitating further research in this area.

## 2 Microbial ecosystem associated with bladder cancer

The onset and progression of bladder cancer arise from the combined influences of multiple factors, including genetic alterations, environmental exposures, chronic inflammation, and immune dysregulation. In recent years, with the ongoing progress of microbiome research, growing attention has been directed toward the potential involvement of the microbiota in the initiation and progression of bladder cancer. The bladder cancer–associated microbial ecosystem primarily comprises the urinary microbiome, the tumor tissue–associated microbiome, and the gut microbiome, which may exert its effects through the gut–bladder axis. These microbial communities and their metabolites may contribute to bladder cancer initiation and progression, shape local and systemic immune responses, and influence therapeutic outcomes and clinical management (**Figure 1**).

**Figure 1:**
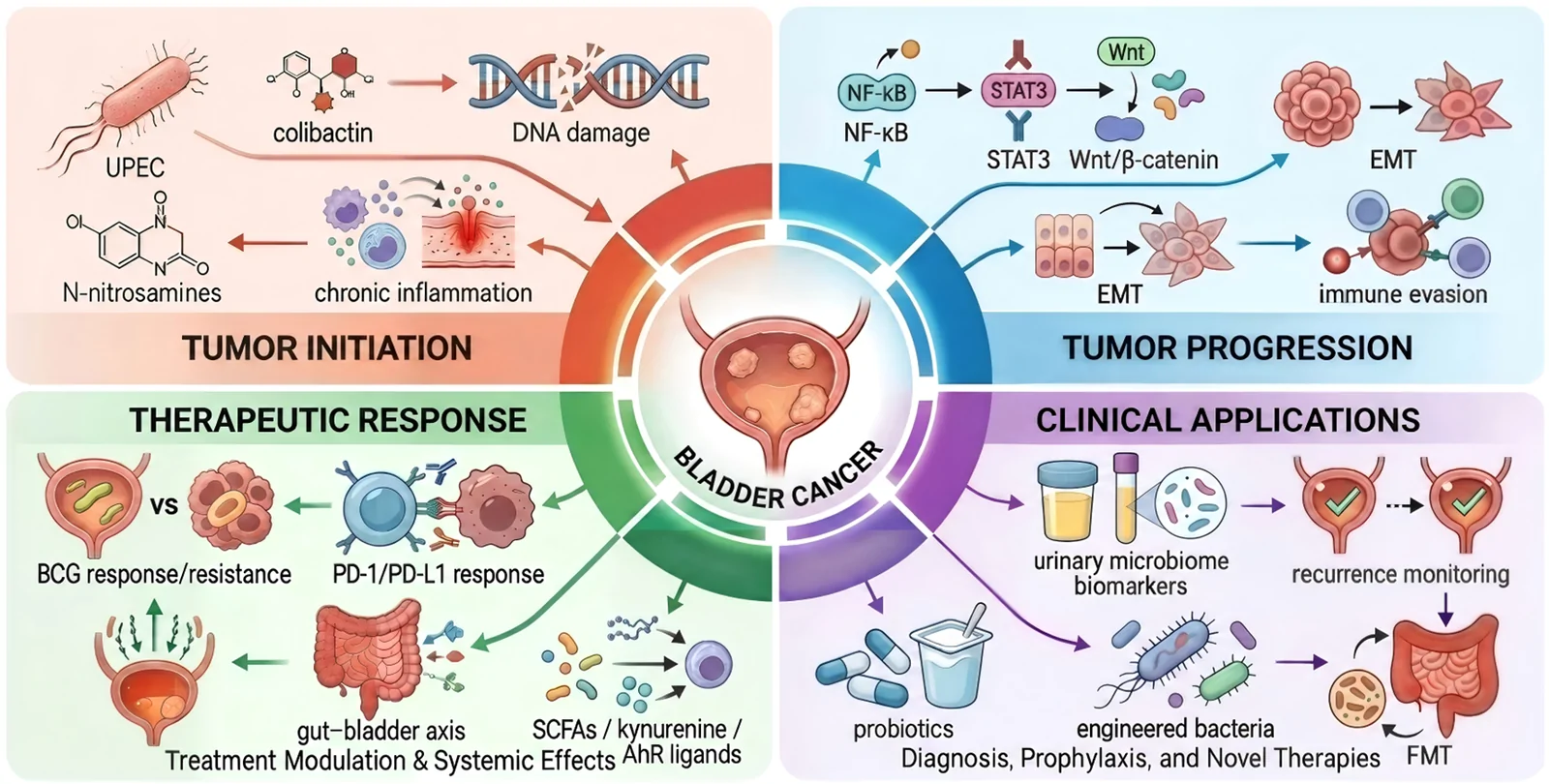
Overview of microbiome-mediated mechanisms in bladder cancer initiation, progression, diagnosis, and therapy. The microbiome may contribute to multiple stages of bladder cancer, including tumor initiation, progression, therapeutic response, and clinical management. During tumor initiation, genotoxic bacteria and their metabolites, such as colibactin and N-nitrosamines, may induce deoxyribonucleic acid (DNA) damage, mutational changes, and chronic inflammation. During tumor progression, microbiota-derived components and metabolites may remodel the tumor microenvironment through inflammatory and oncogenic pathways, including NF-κB, STAT3, AhR, and Wnt/β-catenin signaling, potentially facilitating immune evasion, epithelial–mesenchymal transition, and tumor invasion. In the therapeutic context, the microbiome may influence responses to Bacillus Calmette–Guérin (BCG) immunotherapy and immune checkpoint inhibitors by modulating microbial adhesion, metabolite signaling, antigen presentation, and T-cell activation. Urinary microbiome profiling, probiotics, engineered live biotherapeutic products, and fecal microbiota transplantation (FMT) are being investigated as potential strategies for diagnosis, recurrence monitoring, treatment stratification, and microbiome-targeted intervention; however, most of these applications remain exploratory or preclinical. AhR, aryl hydrocarbon receptor; BCG, Bacillus Calmette–Guérin; DNA, deoxyribonucleic acid; EMT, epithelial–mesenchymal transition; FMT, fecal microbiota transplantation; NF-κB, nuclear factor kappa B; PD-1, programmed cell death protein 1; PD-L1, programmed death-ligand 1; SCFAs, short-chain fatty acids; STAT3, signal transducer and activator of transcription 3; UPEC, uropathogenic *Escherichia coli.* This image was created using BioGDP.

### 2.1 Urinary microbiome

Contrary to the traditional assumption that urine is sterile, the urinary tract of healthy adults harbors a low-biomass but diverse microbial community. The major phyla detected in urine include Proteobacteria, Firmicutes, Actinobacteria, and Bacteroidetes (5). At the genus level, commonly identified bacteria include *Corynebacterium*, *Staphylococcus*, *Streptococcus*, *Lactobacillus*, and *Pseudomonas*, which are considered important for maintaining urinary tract health (5). The composition of the urinary microbiome not only varies among individuals but is also influenced by factors such as sex, age, lifestyle, and environmental exposures.

Sex is one of the major determinants of urinary microbiome composition. Studies have shown that female urine is predominantly characterized by *Lactobacillus* and *Gardnerella*, whereas male urine is more commonly dominated by *Corynebacterium* and *Streptococcus* (9). *Pseudomonas* has also been reported in males but is less frequently observed in females, which may be related to the physiological characteristics of the male urethra and its distinct microbial ecological environment (5). Age may further modify these patterns. In the urine of older individuals, the relative abundance of *Lactobacillus* is decreased. In addition, the *Escherichia* urotype has been linked to older women, whereas the *Gardnerella* urotype is more frequently observed in younger women (5). In addition, factors such as dietary habits, history of antibiotic use, and sexual behavior can also markedly affect the structure of the urinary microbiome.

In addition to biological variation, the observed urinary microbial profile is strongly affected by methodological factors. Midstream and catheterized urine differ in the extent to which they capture urethral, periurethral, and bladder-associated microbial signals and may therefore yield distinct microbial profiles (9). Sample storage and pre-analytical handling (10), deoxyribonucleic acid (DNA) extraction methods (11), and contamination-control and computational procedures (12) can also influence the detected microbial composition. These considerations are particularly important because urine is a low-biomass specimen and is therefore highly susceptible to contamination and technical bias (11, 12).

In bladder cancer, alterations in urinary microbial diversity and taxonomic composition have been reported in comparison with non-cancer controls (13, 14). However, the microbial taxa identified as enriched or depleted are not fully consistent across studies, and urine and bladder tissue may represent distinct microbial niches (13–15). Because urinary microorganisms are located in close proximity to the urothelium, the reported associations between tumor-associated microbial taxa and human beta-defensin levels suggest a potential link between the local microbiota and mucosal immune homeostasis (16). Specifically, increased abundances of *Corynebacterium* and *Staphylococcus* have been observed concurrently with elevated urinary levels of human beta-defensins 2 and 3 (16). Conversely, tumor status and anticancer treatment have also been associated with differences in urinary microbial profiles (17). Therefore, the relationship between urinary microbiome dysbiosis and bladder cancer may be bidirectional; however, its causal and clinical significance requires further investigation in standardized longitudinal studies.

### 2.2 Tumor-associated microbiome

The pathogenesis of bladder cancer is driven by the interplay of multiple factors, including environmental exposures such as smoking and occupational exposure to aromatic amines (18), metabolic abnormalities (19), and pathogenic microbial infections (20). In recent years, accumulating evidence has indicated a link between urinary microbiome dysbiosis and the development of bladder cancer. In a small retrospective cohort, smokers with bladder cancer showed higher urinary abundances of Bacteroidaceae, Erysipelotrichales, and Lachnospiraceae and higher alpha diversity than non-smokers; however, the study design and sample size preclude causal inference (21).

In schistosomiasis-associated bladder cancer, bladder mucosal fibrosis caused by chronic infection may exert a synergistic carcinogenic effect with microbial dysbiosis. *Fusobacterium* and *Sphingomonas* have been implicated in inflammation-associated malignant epithelial transformation through the promotion of N-nitrosamine biosynthesis and activation of the Toll-like receptor 4 (TLR4)/myeloid differentiation primary response 88 (MyD88) signaling pathway (22). Differences among studies may be attributable to differences in sample collection procedures and sequencing depth.

Bladder cancer progression appears to be significantly associated with remodeling of the urinary microbiota (20). Using 16S ribosomal RNA (rRNA) sequencing, Wu et al. identified significant differences in urinary microbial composition between men with bladder cancer and healthy controls, whereas alpha diversity did not differ significantly (14). Because these findings were derived from urine rather than tumor tissue, they should not be interpreted as evidence of intratumoral microbial enrichment. These findings indicate that the carcinogenic process may be driven by the selective expansion of specific pathogenic bacteria rather than by changes in overall microbial diversity (14). Sex-associated differences in urinary microbial composition have been reported; however, the taxa and effect sizes vary across studies and require validation in larger, sex-stratified cohorts (9, 20). *Lactobacillus* has also been reported to be depleted in some bladder cancer cohorts, although its abundance and functional significance remain inconsistent across studies (23).

### 2.3 Gut–bladder axis

The gut–bladder axis refers to the bidirectional interplay between the gut microbiome and bladder homeostasis, mediated by immune, metabolic, and neural pathways (24). Although research in this field is still at an early stage, accumulating evidence suggests that the gut microbiota may influence bladder health not only by modulating systemic immune responses but also by affecting bladder carcinogenesis through microbial metabolites. Microbial translocation and systemic immune or metabolic signaling have been proposed as potential components of the gut–bladder axis. However, direct evidence that gut-derived bacteria reach the bladder and promote bladder carcinogenesis in humans remains limited (25).

At the signaling-pathway level, the gut microbiota and the local bladder microbiota may interact through the gut–bladder axis, potentially contributing to metabolic–immune signaling cascades (**Table 1**). On the one hand, this axis may involve protective metabolic–immune modulation. Short-chain fatty acids (SCFAs) generated by butyrate-producing gut bacteria such as *Roseburia* have been shown to modulate dendritic-cell phenotypes through histone deacetylase (HDAC) inhibition in non-bladder experimental settings (26–28); however, direct evidence that gut-derived SCFAs reach the bladder tumor microenvironment remains limited. Although SCFAs have traditionally been regarded as immunosuppressive factors that promote differentiation of regulatory T cells (Tregs), they can, under co-stimulatory conditions involving T helper 1 (Th1) polarizing signals such as interleukin-12 (IL-12), enhance antigen presentation by dendritic cells and potentiate IL-12 signaling, thereby shifting the immune response toward a Th1 phenotype (26). These findings suggest that the effects of SCFAs are highly dependent on the context and cannot be simply characterized as uniformly immunosuppressive. On the other hand, this axis may also contribute to pro-tumorigenic metabolic imbalance and aberrant signaling activation. During bladder cancer progression, dysregulated tryptophan metabolism may activate aryl hydrocarbon receptor (AhR)-related signaling (29). Evidence from non-bladder studies further suggests that gut microbial alterations may influence tryptophan metabolism and downstream immune responses (30). However, a direct link between gut *Prevotella*, kynurenine metabolism, and Treg or CD8^+^ T-cell responses has not been established in bladder cancer. Meanwhile, this metabolic imbalance is also closely linked to aberrant activation of the Wnt/β-catenin pathway in bladder cancer tissues. Aberrant expression and nuclear translocation of β-catenin have been reported in muscle-invasive bladder cancer (MIBC); however, a direct clinical association between β-catenin activation and the abundance of *Enterococcus faecalis* has not been established. Mechanistically, *E. faecalis* has been proposed to influence inflammatory and Wnt/β-catenin-related signaling (31–33). However, direct evidence that *E. faecalis*-derived cytolysin induces macrophage polarization and drives epithelial–mesenchymal transition in bladder cancer remains lacking.

**Table 1:** Proposed gut–bladder axis-associated metabolic, immune, and signaling pathways relevant to bladder cancer.

Microbial taxa/factors	Primary sources	Major metabolites/virulence factors	Signaling pathways	Immunological/biological effects	Relevance to bladder cancer
*Roseburia*	Butyrate-Producing Gut Bacteria	Butyrate, SCFAs	Inhibition of HDACs; enhanced DC antigen presentation and IL-12 signaling	Remodeling of the immunophenotype of dendritic cells; promotion of Th1 responses under specific conditions	Potentially protective, suggesting context-dependent immunoregulation (34–36)
SCFAs	Gut Microbiota-Derived Metabolites	Butyrate and related metabolites	HDAC inhibition; synergy with Th1-polarizing signals such as IL-12	Traditionally promote Treg differentiation but can favor Th1 responses under specific conditions	Challenge the traditional view that SCFAs are solely immunosuppressive (34–36)
*Prevotella*	Gut microbiota; evidence mainly derived from non-bladder studies	AhR ligand-related metabolites	AhR-related signaling	May modulate Treg and CD8^+^ T-cell responses	Hypothesis-generating; a direct microbiota-mediated relationship has not been established in bladder cancer (37)
Dysregulation of Tryptophan–Kynurenine Metabolism	Gut microbiota and host tryptophan metabolism	Kynurenine-related metabolites	AhR-related signaling; Wnt/β-catenin signaling in tumor tissue	May contribute to immunoregulatory and tumor-associated signaling	Tryptophan and β-catenin signaling have been implicated in bladder cancer, but a direct microbiota-mediated link remains unestablished (37)
*Enterococcus faecalis*	Gut and local microbiota; evidence mainly derived from non-bladder studies	Cytolysin and other microbial products	Proposed inflammatory and Wnt/β-catenin-related signaling	Potential modulation of inflammatory and epithelial signaling	Direct evidence that E. faecalis-derived cytolysin induces macrophage polarization, activates β-catenin signaling, or promotes EMT in bladder cancer is lacking (31–33)
Aberrant Activation of β-Catenin	Aberrant Signaling in Tumor Tissue	—	Wnt/β-catenin	Nuclear translocation and initiation of EMT	Associated with progression and invasiveness in MIBC (31–33)

AhR, aryl hydrocarbon receptor; CD8, cluster of differentiation 8; DC, dendritic cell; EMT, epithelial–mesenchymal transition; GSK-3β, glycogen synthase kinase 3 beta; HDACs, histone deacetylases; IL-12, interleukin-12; MIBC, muscle-invasive bladder cancer; MyD88, myeloid differentiation primary response 88; SCFAs, short-chain fatty acids; STAT3, signal transducer and activator of transcription 3; Th1, T helper 1; TLR4, Toll-like receptor 4; Treg, regulatory T cell.

### 2.4 Comparative discussion of the urinary and gut microbiota

The urinary and gut microbiota represent two distinct but interconnected microbial ecosystems in bladder cancer. The urinary microbiota is anatomically closer to the urothelium and tumor and may therefore better reflect the local bladder microenvironment, whereas the gut microbiota constitutes a larger and metabolically more diverse ecosystem with broader effects on systemic immunity and host metabolism (38, 39). Thus, these two microbial compartments may provide complementary rather than interchangeable information.

Direct comparisons of urinary and gut microbiota in bladder cancer remain limited. Chorbińska et al. analyzed paired urine and stool samples from patients with bladder cancer and healthy controls. Although no significant differences in alpha- or beta-diversity were observed between patients and controls in either compartment, distinct associations with clinical characteristics were identified. For example, *Lactobacillus* was more abundant in urine from patients with a history of Bacillus Calmette–Guérin (BCG) therapy, whereas differences in stool microbiota were associated with tumor grade and sex (38). These findings suggest that urinary and gut microbiota may capture different aspects of bladder cancer biology.

The distinction between the two compartments is also evident in treatment response. Because BCG is administered directly into the bladder, the urinary microbiota may be particularly relevant to local immunotherapy. In a longitudinal study of 12 patients receiving BCG, urinary microbial diversity decreased significantly during the first week of treatment in 10 patients (*P* = 0.021), and baseline microbial composition differed between BCG responders and non-responders (40). By contrast, the gut microbiota has attracted greater attention in systemic immune checkpoint blockade. In the PURE-01 cohort, stool microbiota from 42 patients receiving neoadjuvant pembrolizumab showed distinct microbial profiles between responders and non-responders; *Sutterella* was enriched in responders, whereas *Ruminococcus bromii* was enriched in non-responders (41). In addition, a Chinese metagenomic–metabolomic study identified alterations in gut bacteria and associated metabolites in bladder cancer patients, further supporting the potential systemic metabolic relevance of the gut microbiota (39).

Indirect clinical evidence further supports the importance of the gut microbiota in systemic immunotherapy. In patients with bladder cancer treated with atezolizumab, antibiotic exposure was associated with poorer overall survival and progression-free survival (42). In addition, a multi-institutional prospective cohort including 142 patients with bladder cancer showed that a gut microbiome-based machine-learning model predicted complete response to neoadjuvant chemotherapy more accurately than conventional clinical variables, with corresponding area under the receiver operating characteristic curve (AUC) values of 0.88 and 0.50, respectively. A higher abundance of *Bacteroides* was also associated with a poorer response to neoadjuvant chemotherapy (43, 44). Although antibiotic exposure only indirectly reflects microbial disruption and the chemotherapy-related findings remain associative, these observations support the potential systemic relevance of the gut microbiota without establishing causality.

Overall, the urinary and gut microbiota should be regarded as complementary components of the bladder cancer microbiome–immune network. The urinary microbiota may provide a more direct window into the local bladder environment and intravesical treatment response, whereas the gut microbiota may better reflect systemic immune and metabolic status and responses to systemic immunotherapy (43). Future paired longitudinal studies integrating urine, stool, tumor tissue, and blood samples are needed to determine whether combining local and systemic microbial signatures can improve biomarker development and treatment stratification.

## 3 Mechanistic interplay between the microbiota, bladder carcinogenesis, and immune remodeling

Recent evidence indicates that microorganisms may contribute to the initiation and progression of bladder cancer through multiple pathways, including inflammation, barrier disruption, metabolic reprogramming, DNA damage, and immune reprogramming. Together, these mechanisms constitute a core network through which local and distal microorganisms influence tumor behavior (**Figure 2**).

**Figure 2:**
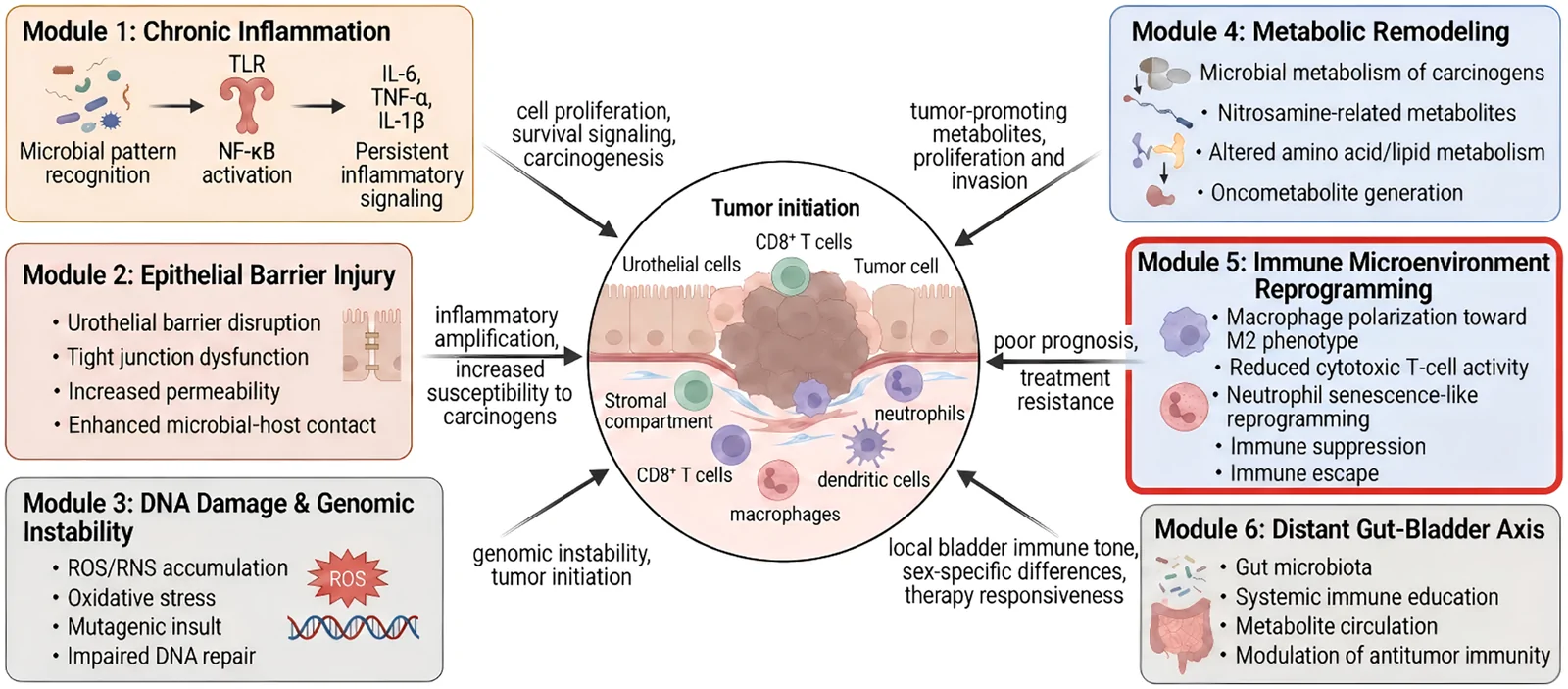
Mechanistic pathways linking microbiota to bladder cancer biology. Microbiota may influence bladder carcinogenesis through multiple non-mutually exclusive mechanisms, including chronic inflammation, epithelial barrier disruption, oxidative DNA damage, carcinogen metabolism, and immune microenvironment remodeling. Local urinary and intratumoral microbes may act directly on the urothelial niche, whereas gut microbiota may exert distal effects through systemic immune and metabolic regulation. Although accumulating data support these associations, causal validation remains limited. CD4, cluster of differentiation 4; CD8, cluster of differentiation 8; DNA, deoxyribonucleic acid; IL-1β, interleukin-1 beta; IL-6, interleukin-6; M2, alternatively activated macrophage phenotype; NF-κB, nuclear factor kappa B; RNS, reactive nitrogen species; ROS, reactive oxygen species; TLR, Toll-like receptor; TNF-α, tumor necrosis factor alpha. This image was created using BioGDP.

### 3.1 Chronic inflammation and innate immune activation

Inflammatory responses may be broadly classified into acute and chronic inflammation. Acute inflammation primarily protects the host against infection through innate and acquired immune responses. In contrast, persistent inflammation is a major contributor to tumor onset and progression. It is characterized by tissue damage, angiogenesis, and fibrosis, and is critical for tumor development and metastasis. Chronic inflammation promotes tumor formation and dissemination by shaping the tumor microenvironment and impairing immune surveillance (45).

Evidence from experimental systems across several cancer types suggests that lipopolysaccharide (LPS) can activate MyD88 through TLR4 and trigger the interleukin-1 receptor-associated kinase 1/4 (IRAK1/IRAK4)–TNF receptor-associated factor 6 (TRAF6)–TGF-β-activated kinase 1 (TAK1)–IκB kinase (IKK) cascade, resulting in IκB-α degradation, nuclear factor kappa B (NF-κB) nuclear translocation, and increased expression of inflammatory mediators such as interleukin-6, interleukin-1 beta, tumor necrosis factor alpha, and cyclooxygenase-2 (COX-2) (46, 47). Microbial stimulation may also promote the recruitment or suppressive activity of myeloid-derived suppressor cells (MDSCs) through chemokine-dependent pathways and the production of reactive oxygen species, peroxynitrite, inducible nitric oxide synthase, and arginase 1 (48, 49). In addition, microbial metabolites such as 4-hydroxyphenylacetic acid have been reported in non-bladder cancer models to activate Janus kinase 2 (JAK2)/STAT3 signaling and recruit polymorphonuclear MDSCs (50). Similarly, the *Fusobacterium nucleatum* FadA–E-cadherin–β-catenin mechanism has been established mainly in gastrointestinal cancer models (51, 52). These findings provide mechanistic plausibility, but their direct contribution to bladder cancer remains to be validated in bladder-specific models and patient samples.

### 3.2 Barrier disruption and epithelial transformation

Epithelial cells play a crucial role in maintaining organ and tissue homeostasis, with the urothelium serving as a key barrier for bladder health. The urothelium forms a physical barrier between urine and the internal environment, and its integrity is maintained by three key components: tight junctions, uroplakin proteins, and the glycosaminoglycan (GAG) layer. In addition, it participates in immune responses and the regulation of the local microenvironment (53). However, persistent pathological stimuli may compromise urothelial integrity and function, thereby contributing to epithelial transformation and tumorigenesis.

Upon prolonged exposure to bacteria, inflammatory mediators, or other harmful stimuli, urothelial cells may progressively lose their normal barrier function. This dysfunction may involve disruption of intercellular junctions and tight-junction proteins such as occludin, thereby compromising urothelial barrier integrity (53). Such barrier disruption may allow toxins, carcinogens, and microorganisms to penetrate the urothelium more readily, potentially exacerbating local immune responses and chronic inflammation. In an experimental urothelial-cell model, *Escherichia coli (E. coli)* exposure was associated with EMT-like changes, including a decrease in the epithelial marker cytokeratin 19 (CK19) from 79% to 51%, an increase in the mesenchymal marker vimentin from 36% to 65%, and significant upregulation of stemness-associated markers, including CD44, SOX2, NANOG, and OCT4 (54). These *in vitro* findings suggest that bacterial exposure may promote epithelial plasticity but do not establish that *E. coli* infection directly induces malignant transformation in patients.

Chronic local inflammation and oxidative stress may promote epithelial dysplasia and contribute to the transition toward a malignant phenotype. Individuals with bladder cancer exhibit significantly elevated levels of malondialdehyde (MDA), a lipid peroxidation product, in both serum and urine, while total oxidative status (TOS) and oxidative stress index (OSI) are also greater than those in healthy controls (55). A possible gut–bladder route has also been proposed. Certain enteric microorganisms, such as *E. coli*, can ascend to the bladder through the urethra and may activate local immune responses and impair epithelial barrier function (25). However, direct evidence that gut microbial dysbiosis promotes bladder carcinogenesis through this route remains limited.

### 3.3 Genotoxic metabolites and DNA damage

One proposed microbiome-associated carcinogenic mechanism in bladder cancer is its potential role as an “endogenous mutagen.” Uropathogenic *Escherichia coli* (UPEC) strains carrying the pks genomic island can synthesize colibactin, a genotoxic polyketide compound. In experimental systems, colibactin acts as a DNA-alkylating agent and can induce DNA double-strand breaks (DSBs) and interstrand crosslinks (ICLs) in urothelial cells (56). Colibactin exposure has also been associated with the single-base substitution signature 88 (SBS88), which is characterized predominantly by T>A and T>C substitutions, particularly within the ATA, ATT, and TTT trinucleotide contexts (57). Although SBS88 has been detected in some urinary tract cancers, most evidence defining this signature has been derived from colorectal tissues and experimental models, and its causal contribution to bladder cancer remains unconfirmed. In a mouse model of urinary tract infection, colibactin-associated DNA damage was detected in keratin 14-positive (Krt14^+^) bladder progenitor cells (56). These findings suggest that recurrent exposure to pks-positive UPEC may contribute to urothelial carcinogenesis; however, its long-term mutational consequences and causal relevance in human bladder cancer remain to be established.

Uropathogenic bacteria carrying nitrate reductase and nitrite reductase, such as *Escherichia coli* (*E. coli*), *Proteus*, *Klebsiella*, and *Staphylococcus*, can sequentially reduce urinary nitrate (NO_3_^-^) to nitrite (NO_2_^-^), which subsequently reacts with amine precursors to form N-nitrosamines. This bacterially mediated nitrosation can occur at urinary pH values below 7.5 and therefore does not necessarily require a strongly acidic environment (58–60). The carcinogenic risk associated with this mechanism may be greater among smokers because tobacco smoke contains aromatic amines, such as 4-aminobiphenyl, and tobacco-specific nitrosamines, including 4-(methylnitrosamino)-1-(3-pyridyl)-1-butanone (NNK), which could interact with bacterial nitrosation and infection-associated inflammation. In a rat model, combined exposure to *E. coli* infection and nitrosamine precursors was associated with a higher incidence of bladder lesions than either exposure alone, accompanied by increased levels of NF-κB, Bcl-2, and IL-6 (58). These preclinical findings support a potential interaction between chronic bacterial infection and chemical carcinogens but do not establish causality in human bladder cancer.

### 3.4 Epigenetic regulation and metabolic reprogramming

Microbiota-derived metabolites and pathogen-associated molecular patterns (PAMPs) may influence epigenetic programs and signaling pathways in tumor and immune cells within the tumor microenvironment (TME) (61, 62). However, direct evidence that these processes act as an oncogenic “switch” in bladder cancer remains limited, because much of the mechanistic evidence has been derived from infection models or other cancer types. Experimental P. aeruginosa studies show that secreted proteases can modify extracellular or immune proteins. LasB exhibits elastolytic activity, whereas alkaline protease can degrade interferon-γ (IFN-γ) (63, 64). However, direct evidence that LasB suppresses IFN-γ-mediated activation of CD8^+^ T cells through cleavage of the IL-6 and TNF-α receptors or induces programmed death-ligand 1 (PD-L1) expression within the bladder cancer TME remains insufficient.

Short-chain fatty acids (SCFAs) are major metabolites generated by gut microbial fermentation of dietary fiber, with acetate, propionate, and butyrate being the principal SCFAs (65). Studies have shown that butyrate can inhibit histone deacetylase (HDAC) activity (66). In bladder urothelial carcinoma cell lines, sodium butyrate has been reported to induce G1-phase cell-cycle arrest and activate the mitochondria-dependent apoptotic pathway (67). These effects appear to be context dependent and have largely been demonstrated in cultured cells; responses may vary according to tumor type and cellular metabolic state (68). Studies conducted primarily in non-bladder cancer experimental models suggest that butyrate may enhance the effector functions of CD8^+^ T cells and chimeric antigen receptor T cells (CAR-T cells) through HDAC-dependent metabolic and epigenetic reprogramming, including increased production of IFN-γ, granzyme B, and TNF-α (69, 70). However, these immunomodulatory mechanisms have not yet been validated in patients with bladder cancer.

Microbial proteases may also alter extracellular-matrix components and host immune recognition (71, 72). In P. aeruginosa infection models, the alkaline protease AprA can degrade monomeric flagellin, thereby attenuating Toll-like receptor 5 (TLR5)-dependent signaling (72). These findings provide a potential mechanism by which bacterial proteases may influence host immunity. However, direct evidence that AprA remodels the bladder cancer TME, disrupts neutrophil extracellular traps (NETs), or facilitates bladder tumor invasion is currently lacking. These observations should therefore be regarded as mechanistically informative but indirect with respect to bladder cancer.

### 3.5 Immune evasion and immune checkpoint regulation

Immune evasion mechanisms in bladder cancer constitute an important area of oncological research. Aberrant activation of the STAT3 pathway has been implicated in bladder cancer immune evasion. In bladder cancer models, downregulation of speckle-type POZ protein (SPOP) increases STAT3 stability, thereby promoting C–C motif chemokine ligand 2 (CCL2) secretion by bladder cancer cells and recruitment of M2-like tumor-associated macrophages (TAMs). In turn, IL-6 secreted by TAMs further activates STAT3, forming a pro-tumorigenic positive feedback loop (73). In addition, studies have shown that the N^6^-methyladenosine (m^6^A) reader protein YTH domain family protein 2 (YTHDF2) suppresses innate immune responses by promoting degradation of retinoic acid-inducible gene I (RIG-I) mRNA. YTHDF2 knockout in bladder cancer cells enhanced the antitumor efficacy of Bacillus Calmette–Guérin (BCG) therapy in orthotopic mouse models of bladder cancer (74). These findings support the involvement of host immune-regulatory pathways but do not establish that resident microbiota drive their dysregulation.

Immune checkpoints, including programmed cell death protein 1 (PD-1), programmed death-ligand 1 (PD-L1), and cytotoxic T-lymphocyte-associated protein 4 (CTLA-4), play essential roles in the regulation of immune responses. Upon engagement with its ligand PD-L1, PD-1 recruits the phosphatase SH2 domain-containing protein tyrosine phosphatase 2 (SHP-2) through its immunoreceptor tyrosine-based switch motif (ITSM), thereby inhibiting T-cell receptor signaling and CD28 co-stimulatory signaling. This process limits the magnitude and duration of T-cell activation, inhibits excessive immune responses, and maintains self-tolerance (75). CTLA-4 primarily functions during the early phase of T-cell activation by competing with CD28 for binding to B7 molecules (CD80/CD86) on the surface of antigen-presenting cells, thereby raising the activation threshold of T cells and negatively regulating T-cell responses (76). Under physiological conditions, these inhibitory pathways are essential for preventing autoimmunity and limiting tissue damage (77).

However, tumor cells can utilize these immune checkpoint pathways to escape immune surveillance. Research has demonstrated that PD-L1 is often overexpressed in various tumor types, including bladder cancer. Upon binding to PD-1 on the surface of T cells, PD-L1 delivers inhibitory signals that can suppress the function of tumor antigen-specific T cells, thereby enabling immune evasion (75, 77). In bladder cancer models, phospholipase A2 group VII (PLA2G7) has been shown to promote PD-L1 expression through activation of the Janus kinase (JAK)–STAT pathway (78). In addition, hypoxia, low pH, and immunosuppressive cells within the tumor microenvironment, such as regulatory T cells and myeloid-derived suppressor cells, further promote PD-L1 expression, thereby aiding in the establishment of an immunosuppressive microenvironment (79).

In addition, the effects of different microbial niches on the initiation and progression of bladder cancer may be mediated through multiple molecular mechanisms. Current studies show substantial heterogeneity in terms of the strength of evidence and the extent of functional validation (**Table 2**).

**Table 2:** Proposed mechanisms through which microbiota may influence bladder cancer.

Mechanistic category	Microbial niche involved	Key pathways or processes	Potential impact on bladder cancer	Current evidence level
Chronic inflammation	Urinary microbiota; mucosa-associated microbiota; intratumoral microbiota	TLR activation, NF-κB signaling, IL-6/TNF-α/IL-1β production, persistent inflammatory tone (25, 43)	May promote urothelial transformation, tumor cell survival, and progression (25, 43)	Moderate associative evidence
Epithelial barrier disruption	Local urinary and mucosal microbiota	Tight junction dysfunction, epithelial permeability increase, enhanced host–microbe contact (25, 43)	May facilitate inflammatory amplification and exposure to carcinogens (25, 43)	Low-to-moderate mechanistic plausibility
Oxidative DNA damage	Local microbiota and inflammation-related processes	ROS/RNS accumulation, oxidative stress, DNA damage, impaired repair (25, 43)	May contribute to genomic instability and tumor initiation (25, 43)	Indirect evidence
Carcinogen metabolism	Gut microbiota	Metabolism of nitrosamine-related or other carcinogenic compounds into tumor-promoting metabolites (25, 43)	May increase systemic or urinary exposure to carcinogenic metabolites (25, 43)	Emerging functional evidence
Metabolic reprogramming	Gut microbiota and intratumoral microbiota	Altered amino acid, lipid, and microbial metabolite networks (25, 43, 80)	May support tumor growth, invasion, and adaptive stress responses (25, 43, 80)	Limited but growing evidence
Immune microenvironment remodeling	Intratumoral microbiota; urinary microbiota; gut microbiota	Macrophage M2 polarization, reduced CD8^+^ T-cell function, neutrophil reprogramming, immune suppression (25, 43, 80)	May contribute to immune evasion and may be associated with prognosis and treatment resistance (25, 43, 80)	Moderate evidence, limited causal validation
Sex-specific immune modulation	Gut microbiota	Sex-dependent immune education, neutrophil senescence-like signaling (25, 43, 81)	May contribute to sex disparities in incidence and outcomes (25, 43, 81)	Emerging mechanistic evidence
Modulation of BCG responsiveness	Urinary microbiota; mucosal microbiota	Altered BCG attachment/internalization, baseline inflammatory state, immune priming (43)	May be associated with differences in BCG response; effects on recurrence risk remain unvalidated (43)	Moderate translational interest, low standardization
Potential modulation of checkpoint blockade	Gut microbiota and systemic immune tone	Antigen presentation, cytokine networks, systemic immune set point (25, 43)	May influence response to immunotherapy (25, 43)	Currently indirect in bladder cancer

BCG, Bacillus Calmette–Guérin; CD8, cluster of differentiation 8; DNA, deoxyribonucleic acid; IL-1β, interleukin-1 beta; IL-6, interleukin-6; M2, alternatively activated macrophage phenotype; NF-κB, nuclear factor kappa B; RNS, reactive nitrogen species; ROS, reactive oxygen species; TLR, Toll-like receptor; TNF-α, tumor necrosis factor alpha.

### 3.6 Bidirectional crosstalk between the microbiome and the local bladder cancer immune microenvironment

The urinary and tumor-associated microbiota form a spatial interface with the urothelium, malignant cells, stromal cells, and infiltrating immune cells. Microbial components and metabolites may regulate epithelial secretion of cytokines, chemokines, and antimicrobial peptides, thereby influencing the recruitment and functional states of neutrophils, macrophages, dendritic cells, Tregs, cytotoxic T cells, and natural killer cells. Conversely, tumor-associated hypoxia, altered nutrient availability, epithelial barrier disruption, and changes in urinary metabolites and antimicrobial peptides may selectively favor or exclude particular microorganisms. Thus, the bladder cancer immune microenvironment and local microbiota may constitute a reciprocally regulated ecological system.

Clinical observations support an association between urinary dysbiosis and the local inflammatory milieu. Wu et al. analyzed urine from 23 treatment-naïve patients with bladder cancer and nine non-cancer controls. Urinary IL-6, IL-8, and IL-10 levels were significantly increased in patients with bladder cancer, while the abundance of *Corynebacterium* correlated positively with IL-6 (*r* = 0.58, *P* < 0.01), IL-10 (*r* = 0.56, *P* < 0.01), and IFN-γ (*r* = 0.35, *P* < 0.05) (82). Notably, the overall urinary microbial structure and cytokine levels remained largely unchanged in ten patients reassessed two to three weeks after tumor resection, suggesting that the inflammatory microbial environment may not simply be a transient consequence of the tumor. However, the small sample size and differences in smoking and urinary tract infection history between groups limit causal interpretation.

More direct evidence connecting intratumoral microbiota with the local immune microenvironment was provided by Chen et al., who integrated paired host transcriptomic and tumor microbial profiles from 401 patients with bladder urothelial carcinoma. Intratumoral *Lachnoclostridium* abundance was associated with increased expression of the chemokines CCL3, CCL4, CXCL9, CXCL10, and CXCL11, as well as greater infiltration of macrophages and CD8^+^ T cells (83). Analyses of two independent immunotherapy cohorts further suggested that these microbiota-associated chemokines were related to treatment response. These findings support a potential microbiota–chemokine–immune-cell axis within the bladder cancer microenvironment. However, because the results were primarily derived from bioinformatic associations, they do not establish bacterial viability, spatial colonization, or a direct causal relationship.

Evidence relating urinary microbiota to adaptive immune phenotypes remains preliminary and sometimes inconsistent. In 40 male patients with non-muscle-invasive bladder cancer, Qiu et al. observed higher urinary microbial diversity in patients who developed recurrence, together with enrichment of *Pseudomonas*, *Staphylococcus*, *Corynebacterium*, and *Acinetobacter*. Higher microbial diversity was associated with increased tumor Ki-67 expression (*P* = 0.0194), but not with intratumoral forkhead box P3-positive (FoxP3^+^) regulatory T-cell (Treg) infiltration (*P* = 0.1653) (84). In another cohort of 28 male patients, the PD-L1-positive group showed greater microbial richness and enrichment of *Leptotrichia, Roseomonas*, and *Propionibacterium*, whereas *Prevotella* and *Massilia* were reduced (85). These findings link microbial features to tumor proliferation and immune phenotypes but do not establish that individual taxa directly induce immune suppression.

Overall, local microbial dysbiosis may reinforce inflammatory, proliferative, or immunosuppressive conditions, while tumor-derived metabolic and immune changes may simultaneously reshape the microbial niche. Because current evidence is predominantly derived from small, cross-sectional cohorts, this bidirectional model remains a working hypothesis. Paired longitudinal sampling of urine and tumor tissue, combined with spatial microbial detection, single-cell immune profiling, metagenomics, and metabolomics, will be necessary to distinguish microbial drivers from tumor-associated consequences.

## 4 Microbiome and treatment response

In the therapeutic landscape of bladder cancer, the microbiome has shifted from being regarded as a mere “bystander” to being recognized as a co-regulator of treatment efficacy. Whether in the context of intravesical BCG instillation or systemic PD-1/PD-L1 immune checkpoint inhibition, the host microbial ecosystem may influence treatment outcomes through complex molecular mechanisms, including modulation of immune activation and metabolic reprogramming.

### 4.1 Bacillus Calmette–Guérin therapy

Intravesical BCG instillation continues to be the standard-of-care treatment for non-muscle-invasive bladder cancer (NMIBC), especially for patients at intermediate and high risk (86). BCG mediates its antitumor activity by activating local immune responses; however, its therapeutic efficacy varies substantially among individuals, and approximately 30–40% of patients are either unresponsive to treatment or experience recurrence (86).

BCG attachment and internalization are important early steps in its antitumor activity. BCG binds to fibronectin, which interacts with α5β1 integrin on urothelial cells, and its internalization involves Rac1/Cdc42-mediated macropinocytosis (87). Whether resident urinary or tumor-associated microbes modify these processes remains incompletely established. In an exploratory study of tumor samples from patients with NMIBC, Knorr et al. observed enrichment of *Lactobacillus* in BCG responders and *Corynebacterium* in non-responders. *In vitro*, coculture with *Lactobacillus crispatus* increased BCG internalization by urothelial carcinoma cells from 10.7% to 17.0% (*P* < 0.0001) (88). These findings suggest that local bacteria may modulate BCG uptake; however, the exploratory cohort and cell-based experiments do not establish a causal effect on clinical treatment outcomes.

Additional bladder cancer-specific observational studies have identified associations between urinary microbial profiles and BCG outcomes. Using 16S ribosomal RNA gene sequencing and functional pathway prediction, Min et al. reported differences in microbial composition and metabolic potential between responders and non-responders. Responders exhibited greater inferred quinolone-biosynthesis potential, while enrichment of *Bifidobacterium breve* and *Bifidobacterium longum* was associated with longer recurrence-free survival (4). Given the limited cohort size and observational design, these taxa and pathways should currently be regarded as candidate biomarkers rather than validated determinants of BCG efficacy. More recently, Liu et al. reported that BCG treatment was associated with alterations in the urinary microbiome and metabolite profiles, including increased propionic acid levels. Subsequent *in vitro* and mouse experiments indicated that propionic acid enhanced BCG-mediated antitumor activity and increased CD4^+^ and CD8^+^ T-cell infiltration (89). These findings provide mechanistic support from bladder cancer cell and mouse models but require prospective clinical validation before propionic acid can be considered a therapeutic adjunct.

Overall, the available evidence supports an association between local microbial features and BCG response, potentially through effects on BCG internalization, epithelial barrier function, mucosal immune homeostasis, microbial metabolism, and local cytokine networks (90). However, direct causal evidence in patients remains limited, and no reproducible microbial signature has yet been established for clinical use. More broadly, microbiota-derived tryptophan metabolites can regulate AhR-dependent immune responses, but evidence directly linking this pathway to BCG resistance in bladder cancer remains insufficient, with much of the mechanistic support derived from experimental models or other cancer types (91–94). Accordingly, AhR-targeted microbial or metabolite interventions should currently be considered hypothesis-generating strategies requiring bladder cancer-specific validation.

### 4.2 Immune checkpoint inhibitors

Immune checkpoint inhibitors (ICIs) have become a crucial therapeutic alternative for patients with advanced bladder cancer (95). However, in clinical practice, resistance continues to represent a major challenge in the treatment of bladder cancer with PD-1/PD-L1 inhibitors, as only approximately 20%–30% of patients achieve durable objective responses, whereas the majority exhibit primary or acquired resistance (96). The gut microbiome has been associated with differences in the therapeutic efficacy of ICIs, although its causal contribution remains unclear (97).

Current immunotherapy for bladder cancer has evolved from single-agent PD-L1 inhibitors, such as atezolizumab, to multimodal combination strategies (79). As discussed in Section 3, experimental studies—many of which were conducted in non-bladder cancer models—suggest that the gut microbiota may modulate ICI activity through pattern-recognition receptor-dependent dendritic-cell activation, IL-12/IL-23 signaling, CD8^+^ T-cell recruitment, and microbial metabolite-mediated regulation of major histocompatibility complex class I (MHC-I) expression (98–101). However, these mechanisms require further validation in bladder cancer. In a small mixed-cancer cohort (n = 50) receiving anti-PD-1 therapy, fecal *Blautia* abundance was higher in responders than in non-responders. Complementary experiments in bladder cancer mouse models suggested that *Blautia coccoides* and its metabolite trigonelline may enhance anti-PD-1 activity by promoting CD8^+^ T-cell infiltration; however, their clinical efficacy in bladder cancer has not yet been established (102).

In an exploratory analysis of pretreatment stool samples from 42 patients with MIBC enrolled in the PURE-01 trial, Pederzoli et al. identified differences in fecal microbial taxa between pathological responders and non-responders. *Sutterella* was enriched in responders, whereas *Ruminococcus bromii* was enriched in non-responders (41). These findings suggest a potential association between the gut microbiome and pembrolizumab response but require validation in larger independent cohorts.

Notably, antibiotic exposure has been associated with decreased efficacy of ICIs (103). A systematic review and meta-analysis by Febriyanto et al. included 13 studies involving 5,095 patients with urothelial carcinoma treated with ICIs, of whom 1,434 (28.0%) were exposed to antibiotics. Compared with patients without antibiotic exposure, those exposed to antibiotics had pooled hazard ratios (HRs) of 1.45 for overall survival (OS; 95% confidence interval [CI], 1.25–1.68) and 1.40 for progression-free survival (PFS; 95% CI, 1.05–1.87) (104). Because antibiotic exposure may also reflect infection, comorbidities, or disease severity, these associations do not establish causality but provide indirect support for a possible relationship between gut microbiome disruption and ICI outcomes.

### 4.3 Other immunotherapeutic approaches

Cytokine-based therapies, especially interferon-based approaches, were among the earliest strategies developed to enhance local immune responses in the bladder. A phase I study by Maeng et al. evaluated an anti-human epidermal growth factor receptor 2 (HER2) autologous dendritic-cell vaccine in 33 patients with HER2-expressing cancers, including two patients with high-risk muscle-invasive bladder cancer treated in the adjuvant setting. The vaccine was generally well tolerated and induced HER2-specific cellular immune responses; however, the very small bladder cancer subgroup precluded conclusions regarding bladder cancer-specific efficacy (105).

In an immunocompetent orthotopic rat model of bladder cancer, Xiao et al. compared intravesical BCG, gemcitabine, interferon-α, and interleukin-2 (IL-2), administered alone or in combination. Monotherapy with BCG, gemcitabine, or interferon-α improved survival and reduced tumor burden compared with the control treatment. Combining high-dose BCG with gemcitabine or interferon-α did not provide additional benefit, whereas low-dose BCG combined with IL-2 improved efficacy (106). These results remain preclinical and require clinical validation.

BCG-induced trained immunity involves epigenetic and metabolic reprogramming of innate immune cells, providing a rationale for the development of modified BCG-based immunotherapeutic strategies (107). Nevertheless, although microbiome-mediated modulation of cytokine-based or dendritic-cell therapies is biologically plausible, direct bladder cancer-specific clinical evidence remains limited.

## 5 Translational applications

Growing evidence suggests that insights into the bladder cancer–associated microbiome are beginning to translate from mechanistic understanding into clinically relevant applications, including biomarker development, therapeutic stratification, and microbiome-targeted interventions.

### 5.1 Diagnostic and monitoring biomarkers

The urinary microbiome has emerged as a highly promising area of research in the diagnosis and management of bladder cancer. Growing evidence indicates that distinct urinary microbial signatures may function not only as diagnostic biomarkers but also as dynamic predictors of recurrence risk and response to immunotherapy. In this context, elucidating the clinical importance of changes in the urinary microbiome could aid in the development of more precise and less invasive methods for diagnosing, monitoring, and tailoring the management of bladder cancer.

#### 5.1.1 As diagnostic biomarkers for bladder cancer

Traditional diagnosis of bladder cancer mainly relies on cystoscopy, which is invasive and uncomfortable, and urine cytology, which has limited sensitivity (108). Owing to its noninvasive nature, urinary microbiome analysis has become a key research focus in the diagnosis of bladder cancer and the monitoring of treatment response (109). The composition of the urinary microbiome differs not only by sex but also between healthy individuals and patients with bladder cancer. Disruption of the urinary microbiome may also induce pro-inflammatory responses by promoting immune cell infiltration, thereby establishing a chronic inflammatory microenvironment that ultimately contributes to the progression of bladder cancer (23). Comprehensive analyses of the urinary microbiome in male individuals with bladder cancer have shown that the abundances of *Acinetobacter* and *Anaerococcus* are significantly increased, whereas those of *Serratia*, *Proteus*, and *Roseomonas* are markedly decreased (14, 36), while *Lactobacillus* appears to be more enriched in healthy controls (110). These findings suggest that bladder cancer-associated urinary dysbiosis may be accompanied by an increase in potentially pathogenic bacteria and a reduction in protective microbial taxa (111) (**Table 3**). Urinary microbial profiles have also been investigated as potential noninvasive diagnostic biomarkers. In a recent study, an extreme gradient boosting (XGBoost)-based urinary microbiota model distinguished patients with bladder cancer from healthy controls, with an AUC of 0.927 in the primary cohort and 0.811 in an external validation cohort (109). However, further validation in large, independent, multicenter cohorts is required before clinical application.

**Table 3:** Principal alterations in the urinary microbiome of patients with bladder cancer.

Microbial taxa	Trend of variation	Clinical significance
*Acinetobacter*	↑	Associated with tumor progression and may serve as a procarcinogenic cofactor (14)
*Fusobacterium*	↑	Associated with accelerated tumor progression and immune evasion, serving as a biomarker of unfavorable prognosis (112)
*Anaerococcus*	↑	Associated with high-grade tumors (36)
*Lactobacillus*	↓	Potentially beneficial taxa; their value as biomarkers of BCG response requires validation (113)
*Serratia*	↓	Enriched in healthy controls (36)
*Proteus*	↓	Enriched in healthy controls (14)
*Roseomonas*	↓	Enriched in healthy controls (14)

BCG, Bacillus Calmette–Guérin; ↑, increased abundance; ↓, decreased abundance.

#### 5.1.2 Applications in postoperative recurrence monitoring

Bladder cancer is marked by a recurrence rate of up to 50%–70%, necessitating lifelong cystoscopic and imaging surveillance (114). Such frequent invasive follow-up not only creates a significant economic burden but also has long-term effects on patients’ quality of life and urinary system function, underscoring the need for noninvasive biomarkers that enable dynamic monitoring and early warning of recurrence.

Cross-sectional studies have identified differences in urinary microbial composition between patients with primary and recurrent bladder cancer. After propensity score matching, one study found lower Chao1 diversity in recurrent bladder cancer (*P* = 0.031) and a significant difference in microbial community structure between primary and recurrent disease (permutational multivariate analysis of variance, *P* = 0.004; R² = 0.025) (114). However, prospective longitudinal evidence demonstrating that urinary microbial alterations precede clinically detectable recurrence remains limited.

Exploratory studies suggest that pretreatment urinary or tumor-associated microbial profiles may be associated with BCG outcomes (40, 88). However, as discussed in Section 4.1, the microbial taxa reported across studies are inconsistent, and no reproducible microbiome-based predictor of BCG response has been clinically validated. Accordingly, these microbial profiles should currently be regarded as candidate stratification biomarkers rather than established tools for postoperative monitoring.

### 5.2 Microbiome intervention and immunotherapy

Microbiome-targeted interventions in bladder cancer mainly include engineered bacteria, commensal bacteria, probiotics, microbial metabolites, fecal microbiota transplantation, and microbiome-based combination strategies with immunotherapy. These approaches may improve treatment efficacy by reshaping the tumor immune microenvironment, enhancing antitumor immunity, and overcoming therapeutic resistance (**Table 4**).

**Table 4:** Concise summary of microbiome-targeted interventions in bladder cancer.

Intervention type	Representative example	Core mechanism	Potential significance
Engineered bacteria	INP-EcN; pGEX-TK-transformed *Bifidobacterium infantis*	Local delivery of IL-12 and anti-PD-1 agents by INP-EcN; HSV-TK/GCV-mediated suicide gene therapy using pGEX-TK-transformed bacteria	Enhance antitumor immunity, promote apoptosis, and improve response to immunotherapy in preclinical models, depending on the construct (115–117)
Commensal bacteria	*Parabacteroides distasonis*	Enhance antitumor immune responses and T-cell infiltration	Increase CD4^+^ and CD8^+^ T-cell infiltration and improve anti-PD-1 efficacy in bladder cancer mouse models (118)
Probiotics	*Lactobacillus rhamnosus* GG; *L. casei* Shirota	Compete with pathogens, produce immunomodulatory metabolites, and regulate oncogenic signaling	Suppress tumor cell growth in experimental models (119, 120)
Microbial metabolites	Propionate, butyrate, AhR agonistic metabolites	Metabolite-dependent modulation of HDAC activity, T-cell polarization, and metabolic reprogramming	May modulate antitumor immunity; clinical benefit in overcoming BCG resistance remains unvalidated (89)
Fecal microbiota transplantation	FMT from ICI responders	May reshape gut microbial composition and modulate systemic antitumor immunity	May improve ICI response; bladder cancer-specific clinical efficacy requires validation (121)
BCG-related microbiome modulation	Beneficial bacteria or metabolite supplementation	May improve mucosal immune homeostasis and strengthen BCG-induced immune activation	Preclinical or exploratory evidence suggests possible modulation of BCG activity; effects on recurrence and clinical response require prospective validation (88, 89)
Targeting putative resistance-associated microbiota	Candidate taxa associated with poor BCG response	Proposed reduction of microbiota-associated immune suppression; effects on BCG uptake require validation	Hypothesis-generating strategy requiring bladder cancer-specific validation (88, 122)
Microbiome-based stratification	Baseline urinary/fecal microbiome profiling	May help predict treatment response and guide personalized intervention	Potential application in precision immunotherapy and risk stratification (88, 123)

AhR, aryl hydrocarbon receptor; BCG, Bacillus Calmette–Guérin; CD4, cluster of differentiation 4; CD8, cluster of differentiation 8; FMT, fecal microbiota transplantation; HDAC, histone deacetylase; HSV-TK, herpes simplex virus thymidine kinase; ICIs, immune checkpoint inhibitors; IL-12, interleukin-12; INP-EcN, interleukin-12 nanoparticle–engineered *Escherichia coli* Nissle 1917; PD-1, programmed cell death protein 1; PD-L1, programmed death-ligand 1; pGEX-TK, recombinant pGEX-5X-1 plasmid encoding herpes simplex virus thymidine kinase; GCV, ganciclovir.

#### 5.2.1 Engineered bacteria

Engineered bacteria can be designed to exploit tumor-associated features, including hypoxia, for localized delivery of therapeutic agents (115, 124). Liao et al. developed INP-EcN, an IL-12 nanoparticle-loaded *Escherichia coli* Nissle 1917, which functions as a “living local drug factory” by continuously biosynthesizing anti-PD-1 and responsively releasing IL-12. This system sequentially promotes T-cell activation and infiltration, thereby markedly reducing tumor growth and metastasis in mouse melanoma models; its efficacy in bladder cancer remains to be established (115).

*Bifidobacterium*, owing to its tumor-hypoxia-targeting properties, has emerged as a potential platform for genetic engineering–based delivery systems (124). A *Bifidobacterium infantis* strain carrying the recombinant plasmid pGEX-TK, constructed by Tang et al., exhibited antitumor activity in a rat model of bladder cancer induced by N-methyl-N-nitrosourea (MNU) by expressing herpes simplex virus thymidine kinase (HSV-TK). Following ganciclovir (GCV) administration, this system induced tumor-cell apoptosis and increased caspase-3 expression (116). Subsequent work implicated both extrinsic and intrinsic apoptotic pathways (117). These findings remain preclinical and require further validation before clinical application.

#### 5.2.2 Commensal bacteria

*Parabacteroides distasonis* may increase ferroptosis sensitivity through its metabolite indole-3-acetic acid (3-IAA), which activates AhR and downregulates fatty acid synthase (FASN) in experimental bladder cancer models (125). In a separate bladder cancer study, oral administration of *P. distasonis* enhanced antitumor immune responses and improved the efficacy of anti-PD-1 therapy in mouse models (118). RNA sequencing demonstrated that *P. distasonis* combined with anti-PD-1 therapy markedly upregulated pathways related to antitumor immune responses in bladder cancer mouse models, including natural killer cell-mediated cytotoxicity, T-cell receptor signaling, and chemokine signaling (all *P* < 0.01) *(*118). Notably, in non-small cell lung cancer (NSCLC) mouse models, *P. distasonis* can enhance CD8^+^ T-cell activity by inducing TAMs to secrete CXCL9; whether this mechanism also operates in bladder cancer remains unclear (126). During PD-1 blockade therapy, co-administration of *Parabacteroides distasonis* increased intratumoral CD4^+^ and CD8^+^ T-cell infiltration without a significant change in FoxP3^+^ Treg density in bladder cancer mouse models (118). These findings do not establish clinical efficacy in patients with bladder cancer.

#### 5.2.3 Probiotics

Probiotics may exert therapeutic effects in bladder cancer. Studies have linked fermented dairy consumption to lower bladder cancer incidence, while some probiotic interventions have shown potential for reducing recurrence (120, 127). Among probiotics, *Lactobacillus* species, in particular, may exhibit antitumor activity by competing against pathogenic strains involved in carcinogenesis or by generating regulatory molecules (23). Animal research has shown that administration of *Lactobacillus rhamnosus* GG can promote tumor regression in bladder cancer models (119). *In vitro* studies have shown that *Lactobacillus rhamnosus* GG and *L. casei* Shirota exhibit growth-inhibitory or cytotoxic effects on bladder cancer cell lines, with comparisons against BCG varying by cell line (120). These findings do not establish clinical superiority over BCG or the efficacy of combining these strains with BCG.

A prospective study from Sweden (n = 82,002), included in the systematic review and meta-analysis by Bermejo et al., showed that daily consumption of at least two servings of fermented milk was associated with a 38% reduction in the risk of bladder cancer compared with no consumption (127). This observational association does not establish a causal protective effect of probiotic supplementation.

#### 5.2.4 Fecal microbiota transplantation

Fecal microbiota transplantation (FMT) represents a broad strategy for microbiome modulation. By transferring a complex fecal microbial community from a screened donor—including bacteria, viruses, and fungi—to the recipient, FMT may reshape microbial composition and function, although engraftment and clinical effects vary (128).

Evidence for FMT in cancer immunotherapy has been derived mainly from non-bladder cancer settings. In preclinical models, transplantation of fecal microbiota from patients who responded to PD-1 blockade into germ-free or antibiotic-treated mice enhanced the antitumor efficacy of PD-1 blockade (99); early clinical studies in immunotherapy-refractory melanoma have also reported preliminary responses (99, 129, 130). Bladder cancer-specific evidence remains preclinical. In MB49 tumor-bearing mice, FMT from healthy donors and subsequent *P. distasonis* mono-colonization enhanced responses to anti-PD-1 treatment (118). A meta-analysis of 10 studies involving 164 patients with solid tumors reported a pooled objective response rate (ORR) of 43% (95% CI, 0.35–0.51) for FMT combined with ICIs. However, the pooled comparison from three randomized controlled trials did not demonstrate a statistically significant improvement in ORR over control treatment (121).

Theoretically, FMT may also influence the bladder immune microenvironment through the gut–bladder axis. Gut microbiota-derived metabolites, including SCFAs, can modulate the tumor microenvironment of distant organs through systemic circulation (31). However, the cited bladder cancer-specific evidence remains preclinical, and clinical efficacy has not been established. Its clinical translation faces numerous challenges, including donor variability, optimization of transplantation protocols, and long-term safety, and therefore warrants further evaluation in clinical trials (128, 131).

## 6 Current limitations and future directions

Although the potential of the microbiota in bladder cancer immunotherapy has become increasingly evident in recent years, substantial limitations remain in the current body of research. The following section summarizes the major limitations of existing studies and outlines future research avenues to further advance the field.

### 6.1 Research heterogeneity and reproducibility issues

Existing studies investigating the microbiome in bladder cancer exhibit substantial methodological and clinical heterogeneity, particularly in study design, cohort size, specimen selection, sample collection, and analytical methods. As noted by Mansour et al., most available studies are cross-sectional, include relatively small sample sizes, and employ different sample collection procedures and sequencing platforms. These inconsistencies limit cross-study comparability and reproducibility and increase uncertainty in the interpretation of the findings (113). Cui et al. further emphasized that microbiome research in urogenital malignancies remains at an early stage. The lack of standardized sampling procedures, analytical approaches, and reporting criteria—including differences in the timing of sample collection, sequencing depth, and data-analysis tools—makes it difficult to interpret findings consistently and draw generalizable conclusions (132). Specimen source represents another major source of heterogeneity. Samples analyzed across studies include midstream urine, catheterized urine, bladder tissue, prostatic fluid, and feces, each of which represents a distinct microbial niche. Mansour et al. compared urinary and bladder mucosa-associated microbiota profiles in patients with bladder cancer and found that *Akkermansia, Bacteroides, Clostridium sensu stricto, Enterobacter*, and *Klebsiella* were significantly enriched in tissue samples compared with urine samples. This finding suggests that urinary specimens may not fully represent the microbial characteristics of the tumor microenvironment (15). Similarly, the review by Heidar et al. indicated that differences in urine collection methods, particularly midstream versus catheterized urine collection, may contribute to inconsistent findings across studies. For example, *Corynebacterium* was reported to be enriched in midstream urine from healthy controls in one study, whereas another study found its enrichment in catheterized urine from patients with bladder cancer (123).

Collectively, these methodological and clinical differences may generate apparent microbial variations driven by technical factors rather than genuine biological differences. They also impede direct comparison and quantitative synthesis across studies, weaken the reproducibility and generalizability of the reported microbial signatures, and limit the strength of the conclusions that can be drawn in this review. Therefore, the current findings should be regarded as primarily qualitative and interpreted with caution until they are validated in prospective multicenter studies using standardized protocols and independent cohorts.

### 6.2 Unclear causal relationship between the microbiome and immunotherapy response

Most reported associations between microbiome features and immunotherapy outcomes are derived from observational cohorts or animal models. Residual confounding, reverse causation, and treatment-induced microbial changes make it difficult to determine whether microbial features are causes, consequences, or correlates of therapeutic response. Therefore, mechanistic studies and longitudinal validation are required to clarify microbiome–immune–tumor relationships in bladder cancer (133).

Longitudinal changes in the microbiome during immunotherapy have not been adequately investigated in bladder cancer. A longitudinal study in patients with advanced melanoma demonstrated changes in gut microbial composition during immune checkpoint blockade (134); however, comparable longitudinal evidence in bladder cancer remains scarce. Consequently, it remains unclear whether the observed microbial alterations precede treatment response or occur as consequences of therapy, antibiotic exposure, infection, or disease progression. Bladder cancer-specific studies incorporating multi-time-point sampling are therefore needed to establish temporal relationships and identify potential intervention windows.

### 6.3 Challenges in the clinical translation of microbiome-targeted therapies

Despite their therapeutic potential, microbiome-targeted interventions face substantial barriers to routine clinical application in bladder cancer. One major challenge is the pronounced interindividual variability of the microbiome. Microbial composition is influenced by age, sex, diet, geographic location, comorbidities, antibiotic exposure, and host immunity. In patients with bladder cancer, repeated urinary instrumentation, tumor resection, intravesical therapy, and systemic anticancer treatment may further alter microbial communities. Longitudinal studies in patients with cancer have also demonstrated marked microbiome fluctuations associated with antibiotic exposure and infectious complications (135). Consequently, a microbial intervention that engrafts or produces an immune response in one patient may be ineffective in another, making it difficult to define a universally beneficial microbial composition or treatment.

A second obstacle is the lack of standardized therapeutic protocols. For probiotics and defined microbial consortia, strain identity, viability, potency, dose, formulation, administration route, and treatment duration may all influence therapeutic effects. FMT additionally requires standardized donor eligibility criteria, pathogen and antimicrobial-resistance screening, stool preparation, storage, and delivery procedures (136). Engineered bacteria introduce further concerns regarding genetic stability, therapeutic-payload expression, biocontainment, kill-switch reliability, and environmental shedding (137). These product-specific differences complicate manufacturing, quality control, cross-trial comparison, and treatment reproducibility.

Safety and regulatory considerations are particularly important because patients with bladder cancer are frequently older, immunocompromised, or affected by treatment-related urothelial injury. Live microbial products may cause bacterial translocation, urinary or systemic infection, excessive immune activation, unintended metabolic effects, or horizontal transfer of virulence and antimicrobial-resistance genes. Donor-derived extended-spectrum β-lactamase-producing *Escherichia coli*, for example, caused bacteremia in two immunocompromised FMT recipients, one of whom died (138). Moreover, the regulatory status of microbiome-based products depends on their intended use and product type. Live biotherapeutic products intended to prevent or treat disease must demonstrate quality, safety, efficacy, manufacturing consistency, and stability before receiving regulatory approval (139). Rigorous product characterization, pathogen screening, traceability, pharmacovigilance, and appropriate biological-containment strategies are therefore essential.

The durability of microbiome-based therapy also remains uncertain. Short-term changes in microbial composition do not necessarily indicate stable engraftment or sustained immune modulation, whereas persistent colonization may produce delayed infection, metabolic effects, or unintended immune consequences. Furthermore, clinical evidence in oncology is currently based mainly on small, early-phase studies. Two proof-of-concept studies combining FMT with anti-PD-1 therapy in melanoma enrolled only 10 and 15 patients, respectively (129, 130), and comparable evidence in bladder cancer remains scarce. Large-scale, well-designed clinical trials with sufficient follow-up are therefore required before the efficacy, safety, durability, and generalizability of microbiome-targeted therapies in bladder cancer can be established.

### 6.4 Future research directions

Future studies should concentrate on several crucial areas: (1) applying high-throughput, multidimensional analytical approaches that integrate microbiome data with host genomic, tumor, and other relevant datasets to clarify the relationship between the microbiota and immunotherapeutic efficacy; (2) developing standardized protocols for microbiome sampling and analysis to ensure comparability across studies; (3) identifying the optimal timing and modes of combining microbiome-based interventions with immunotherapy, while evaluating their clinical feasibility and safety; (4) establishing bladder cancer-specific longitudinal cohorts to determine whether microbiome changes precede or follow treatment response; and (5) strengthening clinical trials of combined microbiome–immunotherapy strategies to assess their real-world therapeutic value in bladder cancer.

In summary, the incorporation of the microbiota into bladder cancer immunotherapy has opened new avenues for cancer treatment, but multiple challenges remain, particularly in clinical translation, personalized intervention, and mechanistic investigation. With ongoing progress in this field, microbiome modulation may become an important component of precision immunotherapy for bladder cancer.

## 7 Conclusion

The urinary, tumor-associated, and gut microbiomes represent spatially distinct but potentially interconnected microbial ecosystems that may influence the initiation, progression, and therapeutic response of bladder cancer. The urinary and tumor-associated microbiomes are more closely associated with the local urothelial and tumor microenvironment, whereas the gut microbiome may exert broader effects on systemic immunity and host metabolism; these microbial compartments should therefore be regarded as complementary rather than interchangeable. Microbial dysbiosis may contribute to bladder carcinogenesis and immune remodeling through chronic inflammation and innate immune activation, disruption of the urothelial barrier, epithelial plasticity, genotoxic metabolite-induced DNA damage, epigenetic and metabolic reprogramming, and altered immune-checkpoint signaling. Within the local tumor immune microenvironment, microbial signals may also influence cytokine and chemokine production and modulate the recruitment or functional states of macrophages, dendritic cells, neutrophils, Tregs, CD8^+^ T cells, and natural killer cells. These interactions are likely bidirectional, as tumor-associated hypoxia, altered nutrient availability, epithelial barrier disruption, host immunity, antibiotic exposure, and anticancer treatments may conversely reshape local and systemic microbial communities.

The microbiome may further affect responses to BCG, immune checkpoint inhibitors, and other immunotherapies by regulating microbial adhesion, innate immune sensing, metabolite signaling, antigen presentation, chemokine-mediated immune-cell recruitment, and T-cell activation. Accordingly, microbial profiles in urine, tumor tissue, and feces have potential value as complementary biomarkers for bladder cancer diagnosis, postoperative recurrence monitoring, and therapeutic stratification. Engineered bacteria, beneficial commensals, probiotics, and fecal microbiota transplantation also represent potential microbiome-targeted strategies for enhancing antitumor immunity. However, most of these applications remain exploratory or preclinical because of methodological heterogeneity, small cohort sizes, inadequate control of confounding factors, contamination risks inherent to low-biomass samples, and the lack of definitive causal and clinical evidence. Key unresolved questions include whether microbial dysbiosis is a driver or consequence of bladder cancer, how the urinary, tumor-associated, and gut microbiomes interact over time, and whether reproducible microbial signatures can predict treatment responses across diverse patient populations.

Future studies should adopt standardized sampling and analytical protocols and establish longitudinal, multicenter cohorts incorporating paired urine, tumor tissue, fecal, and blood samples. Integrating metagenomics, metabolomics, spatial microbial detection, and single-cell immune profiling with functional and mechanistic validation will be necessary to distinguish microbial drivers from tumor-associated consequences. Biomarker-guided clinical trials will also be required to determine the efficacy, safety, durability, and patient-specific applicability of microbiome-based interventions. Advancing from descriptive associations to validated mechanisms and clinically actionable strategies will be essential for incorporating microbiome–immune interactions into the precision management of bladder cancer.
